# Ten simple rules in considering a career in academia versus government

**DOI:** 10.1371/journal.pcbi.1005729

**Published:** 2017-10-05

**Authors:** Philip E. Bourne

**Affiliations:** Data Science Institute and Department of Biomedical Engineering, University of Virginia, Charlottesville, Virginia, United States of America; Whitehead Institute, UNITED STATES

This article is focused on a career point at which a higher degree is in hand—perhaps along with some practical experience—and it is time to make a career decision. One such decision might be between an academic scientific research career versus a non-research career in government service. There are many other opportunities, of course, and industry versus academia has been well covered previously in this series [1]. With federal research funding as limited as it is, early-career scientific researchers are increasingly looking at nonacademic pathways; government service is one option. An example choice might be between accepting a postdoctoral fellowship or tenure track assistant professorship versus becoming a program officer for a funding agency, working in government relations, or working in government policy development. Obviously, these are only a couple of the many career choices available in academia and government. These rules are meant to be as generic as possible by recognizing the broad similarities and differences that exist in the 2 work environments. The rules do not cover the obvious differences, such as the ability to teach in academia but likely not in government.

As indicated, academic research and government service both cover large amounts of career territory. While trying to be as evenhanded as possible between these 2 career paths, undoubtedly, bias stemming from my own experience creeps in, and it is important to understand from where my perspective derives. I have spent most of my career in academia as both a professor and a university administrator. More recently, I spent 3 years in the United States federal government, where I had both an administrative and research role, both in biomedicine. My experience is far from that needed to provide a complete picture of career options. For example, it does not address government service, federal or state, outside of the US. Nor does it truly address the myriad of options outside of working for a government funding agency focused on biomedical research. More problematic is having worked 3 years in government versus over 40 years in academia. Undoubtedly, it is a different article than if I had spent 40 years in government service and 3 recent years in academia. Keeping in mind these limitations and the fact that I have been strongly influenced by the excellent reviews of the first version of this article, what follow are the rules I have to offer, rules which are made as generic as I know how.

Remember also that career options are not for life, and experience in government can be very useful to furthering a career in academia and vice versa. This is something that I can attest to, and which I try to capture.

## Rule 1: Public good means different things

As an academic, I rarely thought about public good, defined as a commodity or service provided without profit to all members of society. Yes, I did my research with the idea of improving the human condition, but that was about it. I gave little thought as to how efficiently and productively I was using public money and the impact that was having on the public. I considered myself central to the enterprise. In government service, the enterprise is central. In government, you are part of a much bigger collective enterprise than the individual research laboratory and its associated discovery. Much of what follows flows from this notion. If you read no further, this defines what is fundamentally different, and it really does take 2 very different personalities. Personally, I would not have been happy in government service if I had not first had an academic career with which I was satisfied. I needed to satisfy the individual before the collective. This sounds selfish, and in some ways it is. This is a good reason, if you are in academia, to respect those in government service around you for their unselfish work! Thinking about the individual versus the collective is also a good basis for really assessing your motives in considering one career path over the other—be honest with yourself.

## Rule 2: Visible rewards and recognition are different and likely on different timescales

In academia, highly cited papers, grants awarded, teaching awards, etc., define success. Again, it is very much centered on the individual or small group. In government, relatively speaking, a new policy, program, etc., likely represents the work of many people. Of course, as humans, we want to be recognized for our efforts. In government, such recognition is not citation and tenure but likely accolades from colleagues, service awards, or promotions. Academia is about individual reward; government service is more about collective reward. Academia is more about broader recognition, including the public, particularly if you make a significant discovery. Achievements in government are generally hidden from the public eye unless you have a very prominent government position—few do. Having said that, it must also be said that academia frequently values some time spent in government—experiences of use to the academic enterprise. And let’s not forget that working for the collective good is a reward in itself for most of us.

Timescales are also different. A paper provides some sense of reward immediately after it is published and your name is on it. Work in government, such as a new policy or program, can take years to be identified as public good, and, as already stated, while understood internally, externally, your name may not even be specifically associated with that outcome.

Again, thinking about the individual versus the collective is also a good basis for really assessing your motives in considering one career path over the other—be honest with yourself.

## Rule 3: Government is more hierarchical than academia

With regard to hierarchy, in my opinion, a star researcher will quickly rise through the ranks and gain tenure. In government, it is more stepwise and experience related. Beyond promotion, reporting structures, while hierarchical in both environments, are adhered to more directly in government. In academia, the hierarchical relationship between a faculty member, department chair, and dean is there as an organizational structure but applied less rigorously than in most branches of government, notably the military.

Consider how much you like working in a structured environment when choosing between academia and government.

## Rule 4: Government offers better job security

Only a fraction of those on the academic ladder gain tenure (i.e., guaranteed salary) and, increasingly, in US parlance, they gain a partial full-time equivalent (FTE), which means that without supplementing your salary with grants, you can’t survive. In other words, given the difficulty today in sustaining grant funding, your academic position is likely not very secure. Thus, depending on how attractive your field of research is to other employment sectors, you could be facing financial hardship. Government jobs often provide more security. Unless the government runs out of money or a department is closed, an unlikely but not unheard of event, you have a job. Thus, there are lots of government jobs that are essentially permanent. For shorter-term definable tasks, contractors are used by the government, for which the government has no obligation after the contract expires. Be sure to understand what type of government job you are applying for.

## Rule 5: Academia generally pays better

The downside of Rule 4 is that government jobs, at least in the US, typically do not pay as well as comparable positions in academia. While true as a general rule, as George Bernard Shaw once wrote, “The only golden rule is that there are no golden rules.” So at the risk of following this statement immediately with a rule, academics tend to have a rather distorted view of what the government can actually pay its employees. This is surprising because you can typically find federal salaries online. The Freedom of Information Act in the US and similar legislation in other countries led to the creation of third-party websites that provide government salary information. This is easily compared with at least state-run academic institutions, which also make this information available. Private institutions are another matter. Explore the possibilities online.

## Rule 6: Both require persistence and patience but in different ways

Red tape plagues any organization of size. The bigger the organization, the more the red tape. Is it proportional to the size of the organization? Let me answer from my own experience. The US National Institutes of Health (NIH) budget is about $32,000,000,000 per year. The research budget of the University of California San Diego is approximately $1,000,000,000 per year. Is the NIH 32 times more bureaucratic? Probably not, but it is significantly more bureaucratic. Some of this serves a purpose. Consider an example. If the NIH makes a policy, it affects the whole of biomedical research in the US and likely beyond. If a Principal Investigator in academia makes a policy, it typically affects little more than that scientist’s lab. NIH policies require legal scrutiny, a period of posting for public comment, and more. In other words, those advocating for the policy need persistence and patience to get it enacted. When that policy finally goes into effect, it has broad-ranging implications. Research obviously requires persistence and patience when, for example, an experiment is not working for unknown reasons, whereas in the case of a government process, the workflow is typically known. The time point to completion in government can be estimated; in academic research, it cannot. In government, the process is often out of the hands of the individual; it is less so in academia. While persistence and patience are required for both academia and a government career, the reasons for persistence and patience are different. I would suggest that it requires a different type of personality for each. Consider how your own persistence and patience match to each environment.

## Rule 7: It is harder to effect change in government, but changes are more likely to persist

As alluded to in Rule 6, an upside of working in government is that, when policies or other actions do get put into place, it is harder for them to be undone. It may require yet a new policy or action to replace the old, which takes time. There needs to be a good reason for the change, and thus, generally speaking, actions taken in government are persistent and hopefully the rewards long standing. Some folks gain satisfaction in knowing this and work well in government. Others are, well, too impatient, as per Rule 6.

Anyone who has sat through an academic faculty meeting might be tempted to say that it is harder to effect change in academia. I would still argue that faculty meeting outcomes generally take less time but have fewer ramifications relative to a government-made decision. Be prepared to work on a longer timescale in government to get your objectives accomplished.

## Rule 8: Working with the private sector is different

Academia and government treat working with the private sector differently, even though, in my experience, government workers are as innovative as their academic counterparts. While academia and government want the translation of research into products, the motivation is different. US academia has the Bayh-Dole Act, which actively encourages the commercialization of university research, and there is more of a direct financial incentive to the inventor and institution. Government workers are far less likely to profit directly from their innovations, and the government has less direct incentive to make money. Companies developing products from government innovations pay taxes, and so money gets fed back into government indirectly rather than directly, as is true of academia.

While perhaps not an innovation per se, government also provides less incentive to publish materials that return royalties, notably books. In the US government, the publisher retains royalties on books, whereas in academia, the author gets the royalties. In summary, if you want to be an entrepreneur, it will be easier in academia.

## Rule 9: Accountability is on a different scale

Government service is generally held to higher ethical standards than academia. This does not mean that government employees are more ethical than their academic counterparts. It simply means that the scale of possible malpractice is different, and the respective academic and government institutions respond to differing degrees. Moreover, an academic institution is responsible to a board of trustees; government is responsible to the public—significantly different levels of accountability. As a result, government responds to even the appearance of malpractice. Consider an example. Being on the scientific advisory board of a company while in academia is typically encouraged. It’s good for the institution to have their faculty recognized in this way. The academic likely benefits from having shares in the company they are helping. Obviously, there are still ethical considerations for academics, such as applying grant monies, originally awarded for a different purpose, to benefit the company they are consulting for. By analogy, a government employee could influence the use of public monies to benefit a company and receive remuneration from that company. Both academic and government scenarios relate to an issue of trust. However, one is damaging to an individual and their academic organization, the other to all of government. Consequently, the ramifications are proportionally different. As a result, government employees are typically subject to tighter rules on what they may and may not do. So for example, if you want to be on scientific advisory boards or consult in a variety of other ways, academia is probably a better choice for you because this is typically not allowed as a government employee.

## Rule 10: Access to resources is different

In general, the government has more access to resources than academia, which are very much “soft money” institutions, where funding is unpredictable and of short duration. More than just a stable source of funding, government has real data, which can be attractive. Think of the National Security Agency (NSA). In the era of data science, characterized by the integration of disparate data sets, government can offer access to data not available to academia to conduct important studies relating to, for example, socioeconomic status and health.

Several years ago, when considering government service while being in academia, I made a pros and cons list to compare the 2 career paths. Hopefully, these rules will help you in creating such a list for yourself. Better still is the hope that others will comment on these rules to provide yet further insights.

Making the career choice presented here is daunting at any stage of one’s career. Furthermore, perspectives may change in one’s career; while academics is more desirable at one stage, service to a community may feel more rewarding at another. It is my hope that these rules will help in weighing trade-offs at any stage of one’s career. Personally, I have thoroughly enjoyed my time in both academia and government, and I have no regrets in switching from academia to government and now back again. But then again, no regrets is my mantra for everything.
